# Population-Based Brain Tumor Survival Analysis via Spatial- and Temporal-Smoothing

**DOI:** 10.3390/cancers11111732

**Published:** 2019-11-05

**Authors:** Chenjin Ma, Yuan Xue, Shuangge Ma

**Affiliations:** 1 School of Statistics, Renmin University of China, Beijing 100872, China; machenjin0310@ruc.edu.cn; 2 Department of Biostatistics, Yale School of Public Health, New Haven, CT 06520, USA; xueyuan115@mails.ucas.ac.cn; 3 School of Mathematics Sciences, University of Chinese Academy of Sciences, Beijing 100049, China

**Keywords:** cancer survival analysis, spatial- and temporal-smoothing, population data, penalized estimation

## Abstract

In cancer research, population-based survival analysis has played an important role. In this article, we conduct survival analysis on patients with brain tumors using the SEER (Surveillance, Epidemiology, and End Results) database from the NCI (National Cancer Institute). It has been recognized that cancer survival models have spatial and temporal variations which are caused by multiple factors, but such variations are usually not “abrupt” (that is, they should be smooth). As such, spatially and temporally pooling all data and analyzing each spatial/temporal point separately are either inappropriate or ineffective. In this article, we develop and implement a spatial- and temporal-smoothing technique, which can effectively accommodate spatial/temporal variations and realize information borrowing across spatial/temporal points. Simulation demonstrates effectiveness of the proposed approach in improving estimation. Data on a total of 123,571 patients with brain tumors diagnosed between 1911 and 2010 from 16 SEER sites is analyzed. Findings different from separate estimation and simple pooling are made. Overall, this study may provide a practically useful way for modeling the survival of brain tumor (and other cancers) using population data.

## 1. Introduction

The analysis of overall survival, progression-free survival, time to metastasis, and other survival outcomes has played an important role in cancer research. The survival models so generated can describe disease paths and facilitate public health and clinical decision-making. Important risk factors identified in such models can lead to a deeper understanding of disease biology and facilitate the identification of high-risk sub-populations and intervention development. Survival modeling has been conducted for most if not all cancer types [1,2,3,4,5], particularly including brain tumor [6,7] which is analyzed in this study. Multiple sources of data have been used in cancer survival modeling. Compared to hospital- and community-based data and data from some other sources, population data can be advantageous by having a smaller possibility of selection bias and higher power, leading to more definitive findings. It is also noted that population data are not problem-free. For example, they may collect limited sets of variables and be less informative. The success of population data-based cancer survival analysis has been well demonstrated [8,9], particularly including that for brain tumor [10]. In terms of statistical analysis techniques, as population data usually have “sample size >> number of covariates”, “classic” survival analysis techniques are usually sufficient. Commonly adopted techniques include the Kaplan–Meier curve, logrank statistic, Cox proportional hazards model, accelerated failure time model, and others. For comprehensive reviews, we refer to Klein and Melvin (2006) [11].

Population data are usually collected from multiple sites spanning over a period of time. In some studies [12,13,14], pooling across multiple time points and/or spatial locations is straightforwardly conducted. For example, in Gnerlich et al. (2007) [15], the analysis of metastatic breast cancer survival is conducted by pooling data from 1988 to 2003 and nine SEER (Surveillance, Epidemiology, and End Results) sites. The validity of such analysis demands homogeneity. In the literature, the variations of important risk factors and magnitudes of their effects in cancer models have been observed [16,17], which are caused by differences in population structure, exposure types/levels, treatment, socio-economic status, cultural factors, and many other factors. As such, simply pooling data across time points and/or locations may mask such important variations or even result in misleading conclusions. To accommodate heterogeneity, analysis has also been conducted for different time points and locations separately. For example, in Pienta, et al. (1995) [18], the analysis of prostate cancer survival is conducted using SEER data limiting to Metropolitan Detroit. One limitation of this approach is that for many cancer types, the sample size for a specific time period and location may be limited, potentially leading to unreliable estimation. For example, in the SEER brain tumor data (details described below), the sample size is only 90 for the 1991–2000 time period and Los Angeles. Cancer is a “slow” disease. Under “ordinary” conditions, variations of the set of important risk factors and their effects are not expected to be “abrupt”, i.e., they should have a certain smoothness property, which potentially enables information borrowing from adjacent time periods and locations to improve power and estimation.

The goal of this study is to conduct cancer survival analysis based on population data, which is a “classic” yet still important problem. Of special interest in this article is the analysis of brain tumor patient survival, which has important public health and clinical implications [19], using SEER data. The key advancement is the development and adoption of a novel spatial- and temporal-smoothing method. Specifically, the heterogeneity across spatial and temporal points is fully accounted for by with location- and time-period-specific survival models. Significantly different from the separate analysis, smoothing of the regression coefficients and models is conducted to enable information borrowing across locations and time periods, which may potentially enhance the power and reliability of analysis. It is noted that the analysis technique can also be applied to other types of cancer and databases other than SEER. As such, this study also has independent methodological value.

## 2. Materials and Method

### 2.1. Materials

A brain tumor is an abnormal mass of tissue within the brain in which cells grow and multiply uncontrollably. More than 150 different brain tumors have been documented, and the two main groups are primary and metastatic. In this article, we focus on the analysis of primary brain tumors, which are categorized as glial or non-glial, as well as benign or malignant [20]. The annual, global, and age-standardized incidence of primary malignant brain tumors is about 3.9 per 100,000 for men and 3.0 per 100,000 for women in 2012 [21]. Between 2002 and 2010, there were 183,740 newly diagnosed cases of malignant brain and CNS tumors in the U.S. [22]. Also in the U.S., the five- and ten-year survival rates are about 29.1% and 25.3% according to the American Cancer Society (ACS) [23]. Brain tumors have been a significant source of cancer-related morbidity and mortality in adolescents and young adults in the U.S. and worldwide.

Data analyzed in this study are obtained from SEER, which is the most comprehensive cancer registry in the U.S. SEER has 18 registries, with one for a different location. It covers approximately 27.8% of the U.S. population. From 1973 to 2015, the SEER registries evolved from nine to 18. Specifically, the nine registries in 1973 are Atlanta, Connecticut, Detroit, Hawaii, Iowa, New Mexico, San Francisco-Oakland, Seattle-Puget Sound, and Utah. The four registries formed in 1992 are San Jose-Monterey, Los Angeles, Rural Georgia, and Alaska. In addition, the five registries formed in 2000 are California, Kentucky, Louisiana, New Jersey, and Greater Georgia. As in the literature [24], brain tumor records are selected using the ICD-O-3 (International Classification of Diseases for Oncology, 3rd edition) codes and CS (Collaborative Stage) schema C71.0–C71.9. In analysis, registries Rural Georgia and Alaska are removed as the sample sizes are too small to generate sensible estimates, leading to a total of 16 registries. In addition, cases diagnosed before 1910 and after 2000 are also removed from analysis because of concerns on sample size.

The following variables have been considered in the literature [25,26] and are included in analysis: age at diagnosis, gender (female, male), marital status (singer, married, others), race (Hispanic white, white, black, others), tumor behavior (benign, malignant, uncertain), tumor grade (others, grade I, grade II, grade III, grade IV,), tumor size, and surgery (no, yes). For categorical variables, dummy variables are created to represent different levels. For each categorical variable, the first category is used as reference. The response variable is overall survival (measured in months) defined as the time length from diagnosis to death. Censoring occurs if patients were alive at the date of last contact. Overall, the analyzed data contains records on 123,571 patients. The year-specific analysis may be challenged by very small sample sizes. To this end, we pool data over ten-year windows. Specifically, the whole time span is divided into nine periods according to the time of diagnosis: 1911–1920, 1921–1930, 1931–1940, 1941–1950, 1951–1960, 1961–1970, 1971–1980, 1981–1990, and 1991–2000. It is acknowledged that this pooling over time may mask certain temporal heterogeneity. However, this may be inevitable and has also been conducted in the literature [14]. It is also noted the proposed analysis can be conducted with other, especially smaller, time window sizes. In fact, with smoothing, it can potentially be less sensitive to the choice of window size than separate estimation. The detailed sample size breakdowns for the 16 registries and nine time periods are provided in Supplementary Table S1 in the Supplementary Materials.

### 2.2. Methods

Assume that data have been collected from p locations and q time intervals (or points). For the ith location and jth time interval, assume that data are available for $n_{i,j}$ independent samples. For the kth sample, we observe $\left(Y_{i,j,k},\delta_{i,j,k},X_{i,j,k}\right)$, where $Y_{i,j,k}$ is the observed time (minimum of the event and censoring times), $\delta_{i,j,k}$ is the event indicator (= 1 if it is an event, and = 0 if censoring), $X_{i,j,k}=\left(x_{i,j,k,1},x_{i,j,k,2},\ldots ,x_{i,j,k,m}\right){\;}^{T}$ is the length-m vector of covariates, and the superscript “*T*” denotes transpose. Without loss of generality, assume that for each location and time interval separately, data have been sorted according to the observed times from the smallest to the largest.

For modeling survival, we adopt the Cox model, which is perhaps the most popular and has been used in a large number of SEER and other population-based studies. For the ith location, jth time interval, and kth subject, the hazard function is

(1) $$h_{i,j,k}\left(t\right)=h_{0}\left(t\right)exp\left({X_{i,j,k}}^{T}\beta_{i,j}\right),$$

where $h_{0}$ is the unknown baseline hazard function, and $\beta_{i,j}={\left(\beta_{i,j,1},\ldots ,\beta_{i,j,m}\right)}^{T}$ is the vector of unknown regression coefficients. It is noted that to fully accommodate the temporal and spatial heterogeneity, the regression coefficients and hence survival models are location- and time-interval-specific. Here the same baseline hazard function is assumed. However, since the baseline hazard function does not show up in estimation (details below), it can vary across time and location. For the ith location and jth time interval, the partial likelihood function is

(2) $$L\left(\beta_{i,j}\right)=\overset{n_{i,j}}{\underset{k=1}{\prod }}{\left[\frac{exp\left(X_{i,j,k}{}^{T}\beta_{i,j}\right)}{\sum_{l\in R\left(t_{i,j,k}\right)}exp\left(X_{i,j,l}{}^{T}\beta_{i,j}\right)}\right]}^{\delta_{i,j,k}},$$

where $R\left(t_{i,j,k}\right)$ is the set of indices at risk at time $t_{i,j,k}$. The log-partial likelihood function is

(3) $$LL\left(\beta_{i,j}\right)=\overset{n_{i,j}}{\underset{k=1}{\sum }}\delta_{i,j,k}\left\{X_{i,j,k}{}^{T}\beta_{i,j}-ln\left[\underset{l\in R\left(t_{i,j,k}\right)}{\sum }exp\left(X_{i,j,l}{}^{T}\beta_{i,j}\right)\right]\right\}.$$

Denote n as the total sample size and β as the vector composed of all $\beta_{i,j}$’s.

We propose the spatial- and temporal-smoothed estimate

(4) $$\hat{\beta }=argmax_{\beta }\left[\overset{p}{\underset{i=1}{\sum }}\overset{q}{\underset{j=1}{\sum }}LL\left(\beta_{i,j}\right)-p\left(\beta ,\lambda_{1},\lambda_{2}\right)\right],$$

where

(5) $$p\left(\beta ,\lambda_{1},\lambda_{2}\right)=\frac{1}{2}\lambda_{1}\overset{p}{\underset{i=1}{\sum }}\overset{q}{\underset{j=2}{\sum }}{\left|\left|\beta_{i,j}-\beta_{i,j-1}\right|\right|}^{2}+\frac{1}{2}\lambda_{2}\overset{q}{\underset{j=1}{\sum }}\overset{p}{\underset{i=1}{\sum }}\overset{p}{\underset{r>i}{\sum }}\omega_{i,r}{\left|\left|\beta_{i,j}-\beta_{r,j}\right|\right|}^{2}.$$

Here $\lambda_{1},\lambda_{2}>0$ are data-dependent tuning parameters and will be chosen using V-fold cross validation. $\omega_{i,r}$ is the weight corresponding to locations i and r and inversely related to the distance between the two locations. In our numerical study, we set it as equal to 1/distance. For inference, the nonparametric bootstrap technique [27] is adopted. Specifically, we sample data with replacement for each time interval and location, and the bootstrap samples have the same sample sizes as the original data. The proposed approach is applied to the bootstrapped data. This procedure is repeated multiple times, and variances of the bootstrap estimates are computed.

Rationale: It is noted that to achieve objectives beyond standard survival analysis, more complex techniques are inevitable. The Cox model is adopted, which can be replaced by other survival models, and the proposed penalized estimation strategy will be directly applicable. The proposed estimation falls into the penalized regularization paradigm, which has been extensively adopted. The key is to achieve information borrowing across time intervals and locations through penalization/smoothing, so as to obtain more accurate estimates for all time and locations. The proposed penalty has two terms. By penalizing their differences, the first term encourages the regression coefficients (and models) between adjacent time intervals to be similar (i.e., changes to be smoother), with the consideration that for a specific location, cancer models do not evolve abruptly. This fused penalization [28] is appropriate when the models can be arranged along a “line”. The second penalty term shares similar spirit and smooths the regression coefficients (and models) between different locations, with the consideration that cancer models for locations that are closer are more similar. As such, the weights are chosen as inversely related to distances. Loosely speaking, this shares a similar spirit as the Laplacian penalization [29]. In contrast to some fused and Laplacian penalization techniques [30], $l_{2}$ penalization is imposed as opposed to $l_{1}$, as in this context sparsity is not of interest. In addition, it can significantly simplify computation. The strategy of smoothing over multiple models has been developed in published epidemiologic studies [31,32]. However, the existing studies are usually limited to one dimension (temporal or spatial) and in different contexts. Two-dimensional smoothing for population-based cancer survival analysis has been lacking.

Remarks: The proposed approach smooths over time and location. It puts no constraint on the relative locations of the data collection sites and is applicable when there are two or more sites. The current formulation is concerned with the SEER data and those alike, which have data from consecutive time intervals. Consider the scenario with missing data, where data are available from time intervals 1, 2, 3, 5, and 6 (that is, no data from time interval 4). In this case, there are two possibilities. The first is to penalize the differences for time intervals 1 and 2, 2 and 3, and 5 and 6. The second is to also penalize the differences for intervals 3 and 5, but imposing an additional weight to reflect that the two models are “farther away”.

#### 2.2.1. Computation

As the overall objective function is continuously differentiable, the Newton–Raphson technique is adopted for optimization. Denote LL(β) as the sum of $LL\left(\beta_{i,j}\right)$’s, and $LL^{\prime }\left(\beta \right)$ and $LL^{\prime \prime }\left(\beta \right)$ as the first and second order derivatives of LL(β), respectively. Conduct Taylor expansion of LL(β) at $\tilde{\beta }$, and keep the first and second order terms:

(6) $$LL\left(\beta \right)\approx LL\left(\tilde{\beta }\right)+{\left(\beta -\tilde{\beta }\right)}^{T}\cdot LL^{\prime }\left(\tilde{\beta }\right)+\frac{1}{2}{\left(\beta -\tilde{\beta }\right)}^{T}\cdot LL^{\prime \prime }\left(\tilde{\beta }\right)\cdot \left(\beta -\tilde{\beta }\right),$$

where

(7) $$LL^{\prime }\left(\tilde{\beta }\right)=\overset{p}{\underset{i=1}{\sum }}\overset{q}{\underset{j=1}{\sum }}\overset{n_{i,j}}{\underset{k=1}{\sum }}\delta_{i,j,k}\left\{X_{i,j,k}-\frac{\sum_{l\in R\left(t_{i,j,k}\right)}X_{i,j,l}\cdot exp\left(X_{i,j,l}{}^{T}\beta_{i,j}\right)}{\sum_{l\in R\left(t_{i,j,k}\right)}exp\left(X_{i,j,l}{}^{T}\beta_{i,j}\right)}\right\}$$

(8) $$LL^{\prime \prime }\left(\tilde{\beta }\right)=-\overset{p}{\underset{i=1}{\sum }}\overset{q}{\underset{j=1}{\sum }}\overset{n_{i,j}}{\underset{k=1}{\sum }}\delta_{i,j,k}\{\frac{\sum_{l\in R\left(t_{i,j,k}\right)}X_{i,j,l}{}^{2}\cdot exp\left(X_{i,j,l}{}^{T}\beta_{i,j}\right)}{\sum_{l\in R\left(t_{i,j,k}\right)}exp\left(X_{i,j,l}{}^{T}\beta_{i,j}\right)}\;-{\left[\frac{\sum_{l\in R\left(t_{i,j,k}\right)}X_{i,j,l}\cdot exp\left(X_{i,j,l}{}^{T}\beta_{i,j}\right)}{\sum_{l\in R\left(t_{i,j,k}\right)}exp\left(X_{i,j,l}{}^{T}\beta_{i,j}\right)}\right]}^{2}\}$$

To improve the stability of computation and reduce cost, we replace $LL^{\prime \prime }\left(\tilde{\beta }\right)$ by a diagonal matrix W(β) whose diagonal elements are the same as $LL^{\prime \prime }\left(\tilde{\beta }\right)$. This strategy has been adopted in the literature when the number of parameters to be optimized is large. Overall, the Newton–Raphson-based optimization proceeds as follows:

(1). Initialize $\tilde{\beta }$. A simple choice of the initial value is zero or the separate estimation (of each location and time interval).

(2). Compute $\hat{\beta }$ as the maximizer of

(9) $$\begin{array}{c} LL\left(\tilde{\beta }\right)+{\left(\beta -\tilde{\beta }\right)}^{T}\cdot LL^{\prime }\left(\tilde{\beta }\right)+\frac{1}{2}{\left(\beta -\tilde{\beta }\right)}^{T}\cdot W\left(\tilde{\beta }\right)\cdot \left(\beta -\tilde{\beta }\right)-p\left(\beta ,\lambda_{1},\lambda_{2}\right), \end{array}$$

using a Newton–Raphson-based technique (details below).

(3). Update $\tilde{\beta }=\hat{\beta }$.

(4). Iterate Steps 2–3 until convergence, which is concluded if the $l_{2}$-norm of the difference between two consecutive estimates is smaller than $10^{-3}$.

In principle, the “standard” Newton–Raphson can be directly applied to maximizing (1). However, it will involve p∗q∗m parameters and inversion of a matrix of this size. We have briefly experimented with this and observed unstable estimation. To tackle this problem, we propose conducting a block-wise optimization in Step (2) of the above algorithm. Specifically, optimization is conducted with respect to a length-m vector of regression coefficients for one time interval and location at a time, holding the other regression coefficients at their current estimated values. This procedure is cycled through all coefficient vectors and repeated multiple times until convergence. 

More specifically, for a specific (i,j) dual, the first order derivative of (1) in Step (2) is computed as

$$LL^{\prime }\left({\tilde{\beta }}_{i,j}\right)+W\left({\tilde{\beta }}_{i,j}\right)\left(\beta_{i,j}-{\tilde{\beta }}_{i,j}\right)$$

(10) $$-\left[2\lambda_{1\beta_{i,j}}-\lambda_{1}\left(\beta_{i,j-1}+\beta_{i,j+1}\right)+\lambda_{2}\left(\beta_{i,j}\sum_{r\neq i}^{p}\omega_{i,r}-\sum_{r\neq i}^{p}\omega_{i,r}\cdot \beta_{r,j}\right)\right].$$

The block-wise solutions can be computed as:

- when 2≤j ≤(q−1),
  (11) $${\hat{\beta }}_{i,j}={\left[W\left({\tilde{\beta }}_{i,j}\right)-I\right]}^{-1}\left[W\left({\tilde{\beta }}_{i,j}\right){\tilde{\beta }}_{i,j}-LL^{\prime }\left({\tilde{\beta }}_{i,j}\right)-\lambda_{1}\left({\tilde{\beta }}_{i,j-1}+{\tilde{\beta }}_{i,j+1}\right)-\lambda_{2}\overset{p}{\underset{r\neq i}{\sum }}\omega_{i,r}\cdot \beta_{r,j}\right],$$
  where I is a m∗m matrix, with diagonal elements equal to $\left(2\lambda_{1}+\lambda_{2}\sum_{r\neq i}^{p}\omega_{i,r}\right)$, and off-diagonal elements equal to 0;
- when j=1,
  (12) $${\hat{\beta }}_{i,j}={\left[W\left({\tilde{\beta }}_{i,j}\right)-I\right]}^{-1}\left[W\left({\tilde{\beta }}_{i,j}\right){\tilde{\beta }}_{i,j}-LL^{\prime }\left({\tilde{\beta }}_{i,j}\right)-\lambda_{1}{\tilde{\beta }}_{i,j+1}-\lambda_{2}\overset{p}{\underset{r\neq i}{\sum }}\omega_{i,r}\cdot \beta_{r,j}\right],$$
  where I has diagonal elements $\left(\lambda_{1}+\lambda_{2}\sum_{r\neq i}^{p}\omega_{i,r}\right)$ and off-diagonal elements 0;
- when j=q,
  (13) $${\hat{\beta }}_{i,j}={\left[W\left({\tilde{\beta }}_{i,j}\right)-I\right]}^{-1}\left[W\left({\tilde{\beta }}_{i,j}\right){\tilde{\beta }}_{i,j}-LL^{\prime }\left({\tilde{\beta }}_{i,j}\right)-\lambda_{1}{\tilde{\beta }}_{i,j-1}-\lambda_{2}\overset{p}{\underset{r\neq i}{\sum }}\omega_{i,r}\cdot \beta_{r,j}\right],$$
  where I has diagonal elements $\left(\lambda_{1}+\lambda_{2}\sum_{r\neq i}^{p}\omega_{i,r}\right)$ and off-diagonal elements 0.

As the objective function is bounded above, and as its value increases in each iteration, the above algorithm is guaranteed to converge. Convergence to the global maximizer can be achieved if all individual models are identifiable. In all of our simulation and data analysis, convergence is achieved within 20 iterations. With simple forms for update, the proposed approach is computationally affordable. The analysis of one simulated dataset (details described in the Supplementary Materials) can be achieved within three minutes on a regular laptop. To facilitate application beyond this study, we have developed an R program and made it available at www.github.com/shuanggema. In the Supplementary Materials, we provide detailed information and Figures S4–S7 on using the R program for data analysis, which may facilitate broader use.

#### 2.2.2. Simulation

We conduct extensive simulation to gain more insights into the proposed analysis and compare with alternatives. Details are presented in Appendix I in the Supplementary Materials. Results of simulation is presented in Tables S7–S8. Visualization of simulation is presented in Figures S1–S3. The key finding is that across the whole spectrum of simulation, the proposed estimation has smaller mean squared errors and smaller variances than the alternative separate estimation, i.e., the overall estimation accuracy is improved.

## 3. Results

**Figure 1:**
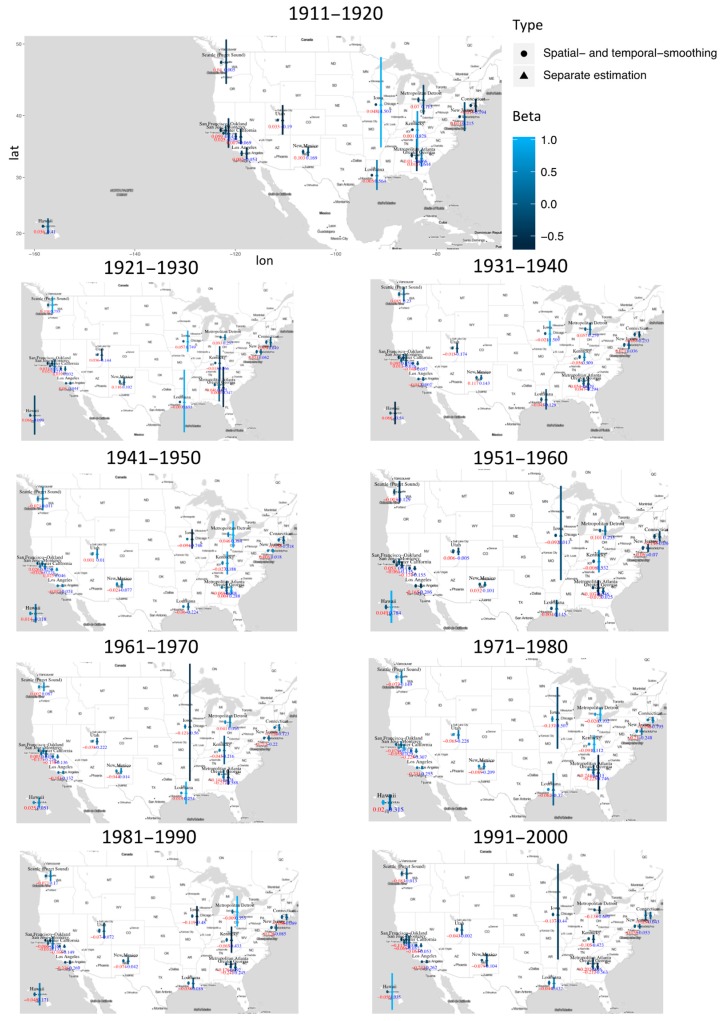
Representative analysis results using the proposed and separate estimations: estimated coefficient (circle or triangle) and its 95% confidence interval (vertical bar) for race (White) at each time interval and location.

**Figure 2:**
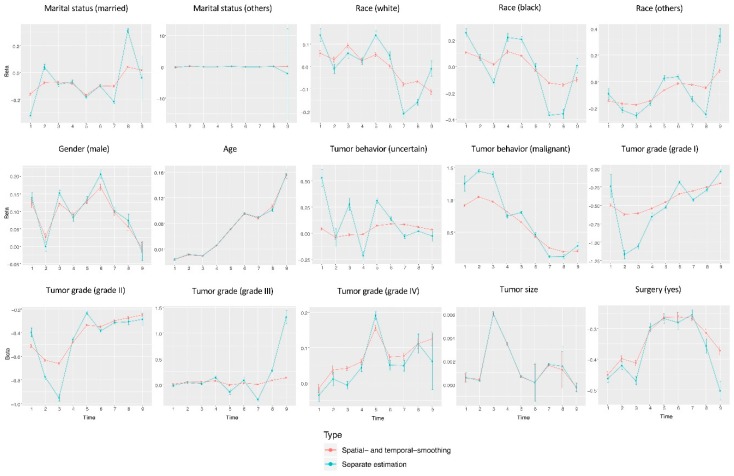
Representative analysis results using the proposed (red) and separate (blue) estimations: estimated coefficient and 95% confidence interval (vertical bar) for each variable at each time interval and location = San Francisco-Oakland.

**Figure 3:**
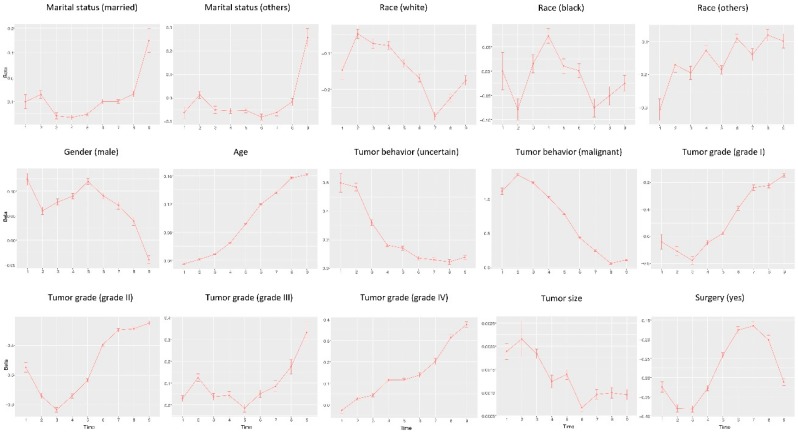
Analysis results for each time interval and by pooling data from all locations: estimated coefficient and 95% confidence interval (vertical bar) for each variable.

**Figure 4:**
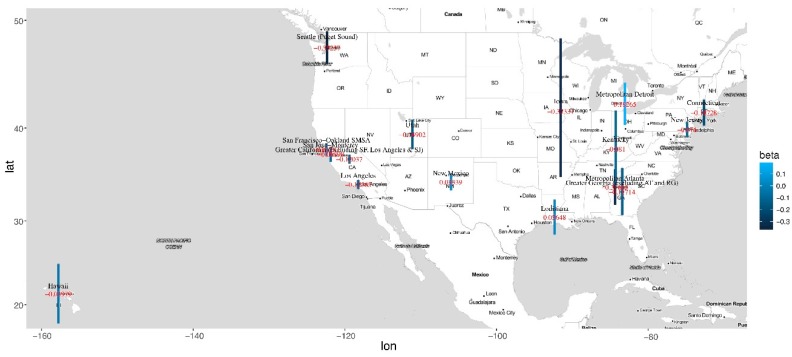
Analysis results for each location and by pooling data from all time intervals: estimated coefficient and 95% confidence interval (vertical bar) for race (White).

Data is analyzed using the proposed approach as well as separate estimation (which conducts the maximum likelihood estimation without penalization under the Cox model for each location and time interval separately). In addition, we also conduct three pooled analysis, under which data from all locations is pooled, data from all time intervals is pooled, and data from all locations and all time intervals is pooled. Detailed estimation results, including the estimated regression coefficients and their standard errors, are provided in Supplementary Tables S1–S6 in the Supplementary Materials. Representative results are also presented graphically. Specifically, in Figure 1, we use covariate race (white) as an example and present the estimates and their 95% confidence intervals for all time intervals and locations using the proposed and separate estimations. In Figure 2, for a representative location (San Francisco-Oakland), we present the estimates and 95% confidence intervals for all covariates as a function of time using the proposed and separate estimations. In Figure 3 and Figure 4, we present the location- and time-pooled analysis results, respectively.

Multiple findings are made. First, the separate estimation (Supplementary Table S3) shows considerable temporal and spatial variations in the estimated coefficients, suggesting the heterogeneity of data. As such, the three sets of pooled analysis may not be sensible. Specifically, averaging over time and/or location may mask important temporal and spatial variations. Consider for example marital status (married). The analysis by pooling data from all locations suggests that since the 1990s, it changes from having a negative correlation with survival to a positive correlation. However, more closely examining data suggests that this change occurs in Connecticut and San Francisco in the 1960s and 1980s, respectively. Another example is race (black). The analysis by pooling data from all time intervals suggests that it is negatively correlated with survival time in San Jose. However, a closer examination of data suggests that before the 1950s, it is positively correlated with survival time in San Jose. There are also other examples of a similar kind. As such, in what follows, we focus on the comparison between the proposed and separate estimations, both of which can sufficiently accommodate heterogeneity.

As can be partly seen from Figure 1, the findings using the proposed approach are mostly qualitatively consistent with those using the separate estimation. Consider for example location = New Jersey, and examine results in Supplementary Tables S2 and S3. Under both approaches, with race (Hispanic white) as reference, race (white) has positive regression coefficients (suggesting a higher risk) until the 1950s, and then the regression coefficients become negative. Our brief literature search suggests that most results from the proposed analysis are consistent with published literature. For example, Villano et al. found that in non-Hodgkin’s lymphoma patients with central nervous system (CNS) tumors, age, sex, and race were all significantly associated with survival. More specifically, older age, male gender, and black race were associated with decreased survival time [33]. Bindal et al. analyzed data on patients treated with surgery as well as those with radiosurgery and concluded that patients undergone surgical treatments survived longer and had a better local control [34]. There are multiple similar published evidence that is consistent with our findings.

The arguably most important finding, which is consistent with that made in simulation, is that the proposed estimates have much tighter confidence intervals, suggesting more accurate estimation. Again, this can be explained by the increased information borrowed from other time intervals and locations. For example, as shown in Figure 1 and Supplementary Table S3, under the separate estimation, the regression coefficient of race (white) in the 1910s in Iowa is 14.503 (sd = 1.196), drops to 0.749 (sd = 0.037) in the 1920s, rises to 11.509 (sd = 0.039) in the 1930s, and drops to −0.746 (sd = 0.030) in the 1940s. Such abrupt changes over time do not seem to be sensible, and there is no literature supporting their validity. In comparison, in Supplementary Table S2 under the proposed estimation, the estimated coefficients (sd) for the same four time periods are 0.048 (0.003), 0.057 (0.004), −0.021 (0.005), and −0.094 (0.004), respectively. For other variables, such improved estimation with tighter confidence intervals is also observed. Tighter confidence intervals can lead to more significant findings. Supplementary Table S2 suggests that most of the results in Figure 1 under the proposed approach are significant expect for time period 1961–1970 and San Francisco-Oakland, 1951–1970 and Seattle-Puget Sound, 1941–1960 and Utah, 1911–1920 and Atlanta, Los Angles, and California, 1931–1940 and San Jose-Monterey, 1911–1920 and 1951–1960 and Kentucky, 1911–1930 and 1951–1960 and Louisiana, 1941–1950 and New Jersey, and 1921–1930 and 1941–1950 and Great Georgia. In comparison, the separate estimation leads to much fewer significant effects.

It is also observed that for some time intervals/locations, the proposed analysis leads to findings different from the separate estimation. For example, for time period 1991–2000 and Connecticut and 1971–1990 and Detroit, the proposed analysis suggests that race (white) has a significant protective effect, but it is a significant risk factor in the separate estimation. A “reversed” conflict exists between time period 1931–1950 and Hawaii and time period 1911–1930 and Utah. As another example, in Figure 2, for covariate race (black), the proposed and separate estimations lead to significantly different estimates at multiple time points. The separate estimation results have a much higher level of variation. As discussed above, cancer is a “slow” disease, and dramatic changes of risk factor effects over time may not be expected. Published literature also has not suggested significant changes in effects (for example, life style, environmental exposures) that may confound with race (black). In addition, in Figure 2, for covariates such as marital status, gender, age, tumor size, and some others, the proposed and separate estimations lead to highly similar results. With the higher stability built in the proposed approach as well as observed in simulation, the estimates generated by the separate estimation that have significant variations are again not expected to be sensible.

There are also findings made with the proposed analysis that have not been sufficiently examined in the literature. For example, published studies suggest that significant racial differences exist in survival (as well as other outcomes such as incidence) for patients with brain tumors. Specifically, five-year survival and incidence are significantly higher in whites [33,35]. Our analysis suggests that race (white) is negatively associated with survival until the 1960s, and then it becomes a positively associated factor. Such a finding has not been well examined in the literature and may be explained by race-related changes in treatment and income around the 1960s.

## 4. Discussion

Population-based survival analysis, for brain tumor as well as other cancer types, is of significant importance. In this article, we have analyzed brain tumor overall survival using the SEER data. As argued above and also in the literature, separate estimation may lead to insufficient power and insensible estimates (for example, abrupt changes without any justification), and pooled estimation may mask important temporal and spatial variations (which have been observed in our data analysis). The proposed approach can directly overcome limitations of the existing methods, and findings mostly consistent with but also different from the existing ones have been made. As established in the literature, such findings can have important public health and medical implications. As it is not the focus of this article, and as there have been multiple published studies on the overall survival of brain tumor patients, interpretations of the findings are not extensively pursued. Like in published studies, there is a lack of gold standard to validate the findings. The satisfactory results observed in simulation and the fact that the data analysis results are “as expected” (in particular the tighter confidence intervals) provide confidence to the findings. It is noted that the proposed approach is not limited to brain tumor and SEER data. It will be well applicable to other cancers and other databases. In addition, it will also be applicable when there exists only one dimension (spatial or temporal).

## 5. Conclusions

Survival analysis has essential importance for brain tumor and other cancers. This study has developed a new analysis technique, which improves estimation accuracy by “borrowing information” spatially and temporally. The proposed method is built on the penalization technique and has a sound statistical basis. Simulation and the analysis of SEER data demonstrate its satisfactory performance. It is expected that it can also improve estimation for other cancers and databases and will have broad applications. 

## Supplementary Materials

The following are available online at https://www.mdpi.com/2072-6694/11/11/1732/s1. Detailed simulation and estimation results and demonstration of data analysis using the R code referred to in the main text are available in the document named “Supplementary Materials”. Figure S1: Simulation. Left panel: true regression coefficients as a function of space coordinates (x and y) generated from a part of the 1/8-spherical surface; Middle panel: true regression coefficients as a function of space coordinates (x and y) generated from a part of the 1/2-spherical surface; Right panel: true regression coefficients as a function of time (black: wave-based; red: monotonically increasing; and blue: concave); Figure S2: Simulation: Case 1, Scenarios 1–3 (left to right) with n = 400. True and estimated coefficients and their point-wise 95% confidence intervals using the proposed and separate estimations; Figure S3: Simulation: Case 1, Scenario 2 (left) and Case 2, Scenario 2 (right) with n = 400. True and estimated coefficients and their point-wise 95% confidence intervals using the proposed and separate estimations; Figure S4: Structure of the brain tumor brain dataset in R; Figure S5: Functions loaded in R; Figure S6: Visualization of an example of marital status (married) at each time interval and location=San Francisco-Oakland; Figure S7: Visualization of an example of marital status (married) for the time interval 1911–1920 and each location; Table S1: Data analysis: sample size for each time interval and location; Table S2: Data analysis: estimated coefficients and standard deviations using the proposed approach; Table S3: Data analysis: estimated coefficients and standard deviations using the separate estimation; Table S4: Data analysis: estimated coefficients and standard deviations by pooling all time intervals and locations; Table S5: Data analysis: estimated coefficients and standard deviations by pooling all locations; Table S6: Data analysis: estimated coefficients and standard deviations by pooling all time intervals; Table S7: Simulation I: mean squared errors (MSE) and variance (Var) of the proposed and separate estimations; Table S8: Simulation II: mean squared errors (MSE) and variance (Var) of the proposed and separate estimations.
