# Supplementary material for: Population-Based Brain Tumor Survival Analysis via Spatial- and Temporal-Smoothing
Source: Cancers (Basel). 2019 Nov 5;11(11):1732. doi: 10.3390/cancers11111732 (PMC6895900; doi:10.3390/cancers11111732)
Supplement: Supplementary file 1 [file cancers-11-01732-s001.zip › cancers-613455-SI/cancers-613455-Supplementary Materials final2 .docx]

Population-based Brain Tumor Survival Analysis via Spatial- and Temporal-Smoothing

Chenjin Ma, Yuan Xue and Shuangge Ma

Appendix I: Simulation

We conduct simulation to evaluate performance of the proposed spatial- and temporal-smoothing and compare with the separate estimation, which conducts estimation under the Cox model without any penalization for each time interval and location separately. A variety of simulations settings are considered. Specifically, we consider two levels of “signals”. Under Simulation I, the regression coefficients range from 0 to 1; and under Simulation II, they range from 0.8 to 1.3. Under all settings, there are ten covariates. Multiple covariate distributions are considered. Under Case 1, covariates are generated from a multivariate normal distribution with marginal means 0, marginal variances 1, and an auto-regressive correlation structure with parameter 0.5 (that is, covariates $k$ and $l$ have correlation coefficient ${0.5}^{|k-l|}$). Under Case 2, covariates are first generated as under Case 1. Then half of the covariates are dichotomized to generate Bernoulli distributions. The probabilities of success are around 0.3. Under Case 3, covariates are first generated as under Case 1. Then all covariates are dichotomized to generate Bernoulli distributions as under Case 2. Under each case, we consider six Scenarios (Scen) of spatial and temporal relationships among coefficients/models. Specifically, we “decompose” the regression coefficients into a spatial and a temporal component. On a (0,1)*(0,1) square, we first select nine locations, whose spatial distributions have properties comparable to the nine SEER locations. Then the spatial components of the regression coefficients are obtained from either 1/8 of a spherical surface (left panel of Figure S1) or ½ of a spherical surface (middle panel of Figure S1). Then for ten time intervals (points), the temporal components of the regression coefficients are obtained from one the three curves in the right panel of Figure S1, including a wave-based, a monotonically increasing, and a concave curve. The spatial and temporal coefficients are then multiplied together, leading to a total of six scenarios. The coefficients are further multiplied with the same constants to achieve the desired ranges. The event times are generated from Cox models with baseline hazard $h_{0}\left( t \right)=\left( t-0.5 \right)^{2}$. Censoring times are independently generated from exp(0.12). In addition, to mimic the SEER data, censoring times are truncated. The overall censoring rates are about 20%. At each time interval and location, we consider sample size = 200, 400, 1000, and 2000. For each setting, 200 replicated are generated, and summary statistics are computed.


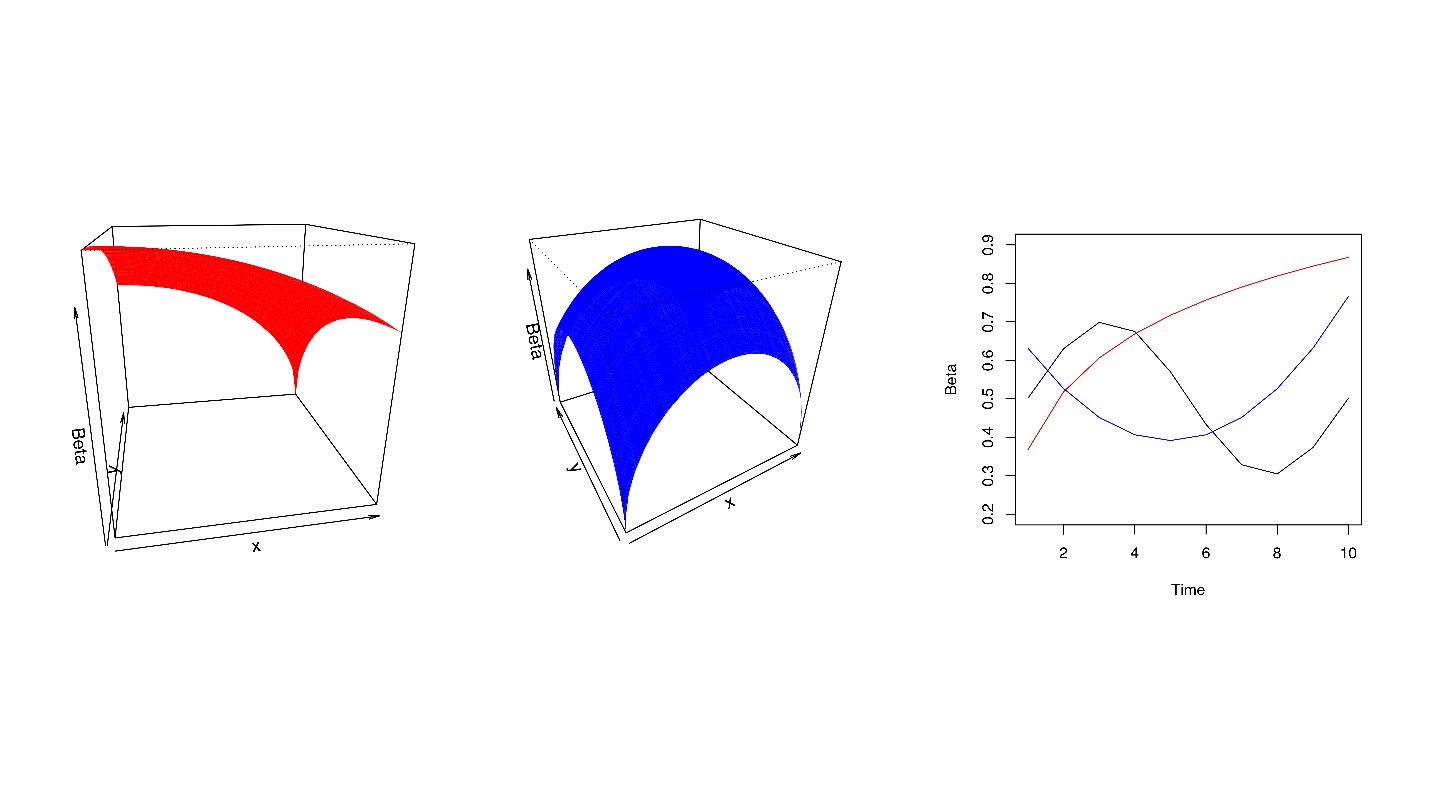


**Figure 1.** Simulation. Left panel: true regression coefficients as a function of space coordinates (x and y) generated from a part of the 1/8-spherical surface; Middle panel: true regression coefficients as a function of space coordinates (x and y) generated from a part of the 1/2-spherical surface; Right panel: true regression coefficients as a function of time (black: wave-based; red: monotonically increasing; and blue: concave).

The main goal of the proposed analysis approach is to improve estimation via information borrowing/smoothing. As such, we focus on the evaluation of estimation accuracy. In Tables A1 and A2, we present the mean and variance of MSE (mean squared errors) for the 10*9*10 regression coefficients, and for the proposed approach and separate estimation. In addition, in Figure S2, for a representative of each temporal curve shape, Case 1, Scenarios 1–3, and sample size = 400, we present the mean estimates and their point-wise 95% confidence intervals. Here we examine the temporal dimension and sum over locations. In Figure S3, we consider Case 1, Scenario 2 (left panel, the spatial components of the regression coefficients are from the 1/8 spherical surface, and the temporal components are from the monotonically increasing curve) and Case 2, Scenario 2 (right panel), with sample size = 400. We also present the mean estimates and their point-wise 95% confidence intervals. Here we examine the spatial dimension and sum of time intervals.


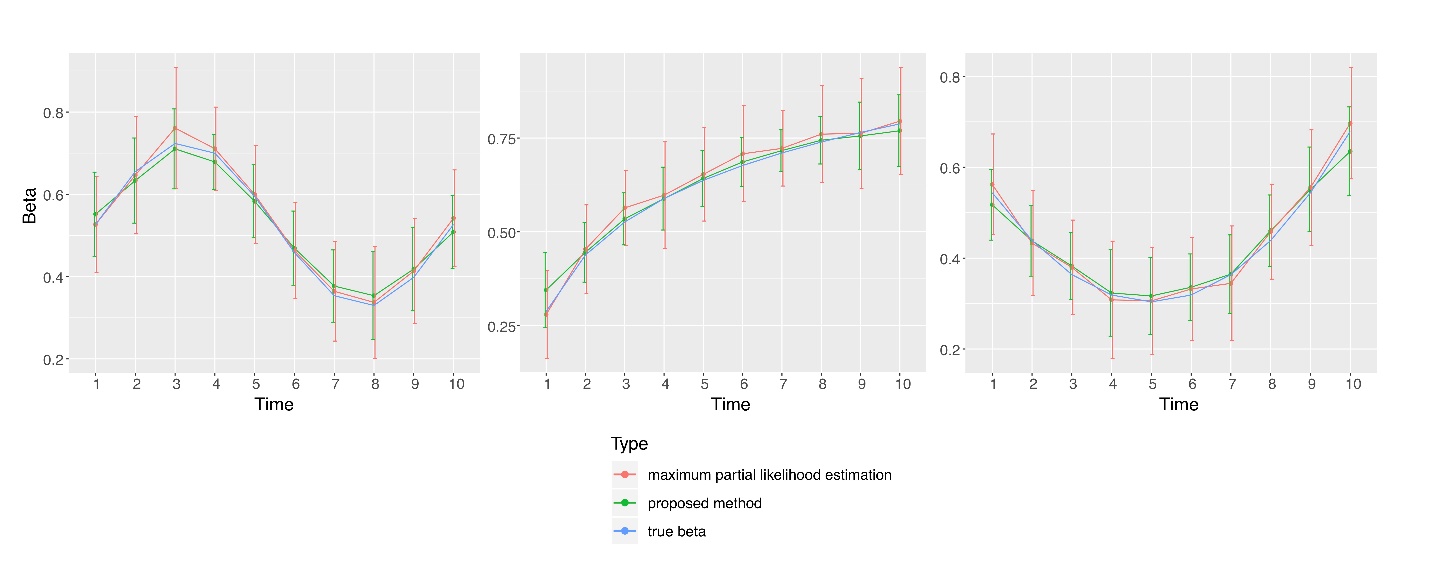


**Figure 2.** Simulation: Case 1, Scenarios 1–3 (left to right) with n = 400. True and estimated coefficients and their point-wise 95% confidence intervals using the proposed and separate estimations.


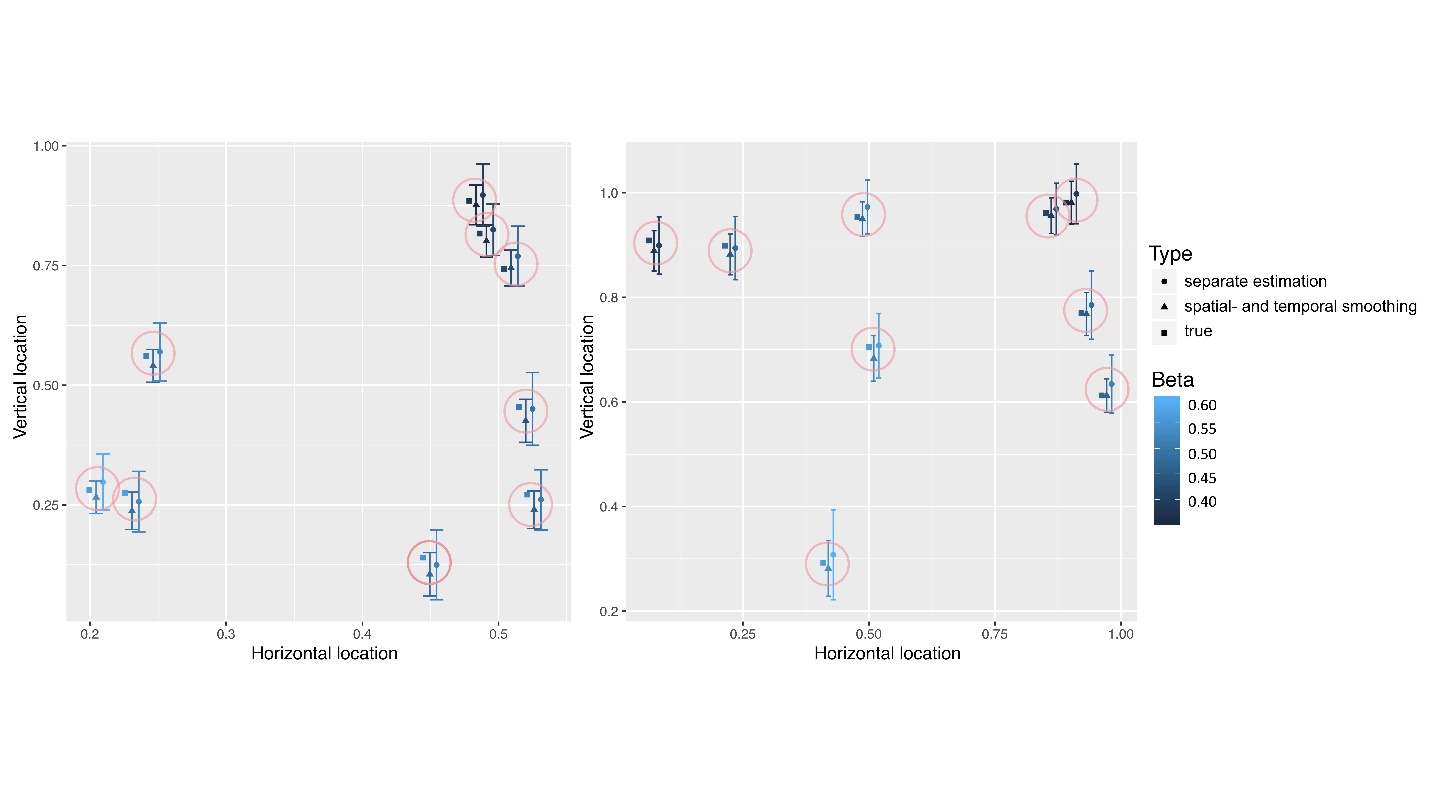


**Figure 3.** Simulation: Case 1, Scenario 2 (left) and Case 2, Scenario 2 (right) with n = 400. True and estimated coefficients and their point-wise 95% confidence intervals using the proposed and separate estimations.

All simulation scenarios lead to the same conclusion that the proposed smoothing approach has improved estimation accuracy. For example, for Simulation I, Case 1 with sample size = 200, the mean MSEs of the proposed approach range from 2.958 (Scenario 5) to 4.718 (Scenario 1), where as those of the separate estimation range from 8.118 (Scenario 6) to 9.283 (Scenario 5). The MSEs of the proposed approach also have smaller variances, suggesting more stable estimation. When covariate distributions get “less continuous” (from Case 1 to 3), estimation gets more challenging, and performance of both approaches deteriorates. Here the superiority of the proposed approach gets more prominent. Consider for example Simulation 1, Case 3 with sample size = 200. The mean MSEs of the proposed approach range from 6.180 to 7.294, whereas those of the separate estimation range from 162.860 to 246.972. This significant improvement is sensible. There is “less information” with discrete covariates. As such, there is a stronger demand for information borrowing across time and location. When sample size increases to 2,000, the proposed approach still has favorable or comparable performance. However, the level of improvement decreases. In both Tables A1 and A2, for Case 1 with sample size = 2,000, the proposed approach only has slight advantage. This is also sensible. For “easy data” with a large sample size (note that here the total sample size is 2,000*9*10), there is “sufficient information”, making information borrowing not necessary. It is noted that for brain tumor as well as quite a few other cancer types in SEER, sample sizes for multiple time intervals and locations are considerably smaller than 2,000.

**Description of Supplementary Tables S1–S6**

**Table S1:** Data analysis: sample size for each time interval and location.

**Table S2**: Data analysis: estimated coefficients and standard deviations using the proposed approach.

**Table S3**: Data analysis: estimated coefficients and standard deviations using the separate estimation.

**Table S4:** Data analysis: estimated coefficients and standard deviations by pooling all time intervals and locations.

**Table S5:** Data analysis: estimated coefficients and standard deviations by pooling all locations.

**Table S6:** Data analysis: estimated coefficients and standard deviations by pooling all time intervals.

**Table S7:** Simulation I: mean squared errors (MSE) and variance (Var) of the proposed and separate estimations;

**Table S8:** Simulation II: mean squared errors (MSE) and variance (Var) of the proposed and separate estimations;

**Table S7.** Simulation I: mean squared errors (MSE) and variance (Var) of the proposed and separate estimations.

|  |  | **Sample size = 200** | | | | **Sample size = 400** | | | | **Sample size = 1000** | | | | **Sample size = 2000** | | | |
| --- | --- | --- | --- | --- | --- | --- | --- | --- | --- | --- | --- | --- | --- | --- | --- | --- | --- |
|  |  | **proposed** | | **separate** | | **proposed** | | **separate** | | **proposed** | | **separate** | | **proposed** | | **separate** | |
| **Case** | **Scen** | **MSE** | **Var** | **MSE** | **Var** | **MSE** | **Var** | **MSE** | **Var** | **MSE** | **Var** | **MSE** | **Var** | **MSE** | **Var** | **MSE** | **Var** |
| 1 | 1 | 4.718 | 3.607 | 8.931 | 8.452 | 3.494 | 1.652 | 3.933 | 3.793 | 1.173 | 1.050 | 1.376 | 1.395 | 0.618 | 0.567 | 0.695 | 0.687 |
|  | 2 | 4.580 | 3.417 | 9.049 | 8.604 | 2.215 | 1.732 | 4.344 | 4.169 | 1.347 | 1.211 | 1.515 | 1.165 | 0.729 | 0.461 | 0.749 | 0.746 |
|  | 3 | 4.065 | 2.965 | 8.882 | 8.392 | 2.823 | 1.473 | 3.745 | 3.658 | 1.226 | 1.076 | 1.362 | 1.352 | 0.572 | 0.522 | 0.654 | 0.651 |
|  | 4 | 4.313 | 3.216 | 8.530 | 8.154 | 2.716 | 2.042 | 3.732 | 3.646 | 1.287 | 1.276 | 1.354 | 1.339 | 0.613 | 0.638 | 0.718 | 0.652 |
|  | 5 | 2.958 | 2.205 | 9.283 | 8.775 | 2.057 | 1.803 | 3.931 | 3.822 | 1.190 | 1.160 | 1.474 | 1.449 | 0.550 | 0.533 | 0.711 | 0.705 |
|  | 6 | 3.642 | 2.648 | 8.118 | 7.853 | 2.170 | 1.670 | 3.555 | 3.500 | 1.024 | 0.863 | 1.313 | 1.299 | 0.545 | 0.497 | 0.632 | 0.631 |
| 2 | 1 | 9.814 | 6.234 | 256.934 | 256.576 | 10.155 | 5.856 | 116.011 | 113.179 | 3.913 | 2.987 | 28.685 | 28.676 | 2.455 | 1.944 | 15.812 | 15.807 |
|  | 2 | 10.026 | 6.262 | 257.273 | 256.601 | 6.821 | 4.876 | 492.627 | 491.916 | 4.009 | 2.999 | 34.959 | 34.936 | 2.872 | 2.772 | 23.814 | 23.791 |
|  | 3 | 8.547 | 6.089 | 312.067 | 311.937 | 6.140 | 4.546 | 137.243 | 136.915 | 3.541 | 2.928 | 41.136 | 41.067 | 2.408 | 1.915 | 21.825 | 21.800 |
|  | 4 | 9.823 | 6.267 | 335.753 | 335.177 | 6.679 | 4.698 | 119.999 | 119.908 | 4.047 | 3.420 | 61.184 | 61.142 | 2.355 | 1.877 | 15.213 | 15.194 |
|  | 5 | 7.394 | 5.196 | 352.061 | 351.947 | 4.979 | 3.665 | 111.042 | 110.936 | 2.795 | 2.259 | 41.894 | 41.867 | 1.860 | 1.619 | 18.182 | 18.145 |
|  | 6 | 8.842 | 6.126 | 386.692 | 386.549 | 5.999 | 4.337 | 143.828 | 143.156 | 3.379 | 2.588 | 70.924 | 70.851 | 2.061 | 1.636 | 22.736 | 22.719 |
| 3 | 1 | 7.294 | 4.619 | 246.972 | 247.407 | 4.824 | 3.518 | 108.333 | 108.076 | 2.467 | 1.962 | 58.686 | 58.277 | 1.524 | 1.255 | 19.143 | 19.133 |
|  | 2 | 5.446 | 3.930 | 192.424 | 191.729 | 3.942 | 2.641 | 104.083 | 103.560 | 1.848 | 1.455 | 48.435 | 48.413 | 1.168 | 1.042 | 18.673 | 18.630 |
|  | 3 | 6.580 | 4.565 | 165.326 | 164.458 | 4.323 | 3.443 | 96.538 | 96.054 | 2.250 | 1.747 | 46.337 | 46.297 | 1.330 | 1.252 | 17.118 | 17.106 |
|  | 4 | 6.180 | 4.117 | 220.910 | 219.376 | 3.957 | 2.643 | 102.503 | 101.506 | 1.866 | 1.415 | 33.790 | 33.726 | 1.143 | 1.029 | 16.378 | 16.357 |
|  | 5 | 6.512 | 3.961 | 162.860 | 161.436 | 3.600 | 3.020 | 108.495 | 107.479 | 2.158 | 1.897 | 36.912 | 36.897 | 1.095 | 1.008 | 17.529 | 17.515 |
|  | 6 | 7.098 | 5.117 | 167.861 | 166.668 | 4.044 | 2.937 | 105.497 | 104.584 | 2.489 | 2.036 | 46.029 | 45.967 | 1.539 | 1.433 | 16.508 | 16.496 |

**Table S8.** Simulation II: mean squared errors (MSE) and variance (Var) of the proposed and separate estimations.

|  |  | **Sample size = 200** | | | | **Sample size = 400** | | | | **Sample size = 1000** | | | | **Sample size = 2000** | | | |
| --- | --- | --- | --- | --- | --- | --- | --- | --- | --- | --- | --- | --- | --- | --- | --- | --- | --- |
|  |  | **proposed** | | **separate** | | **proposed** | | **separate** | | **proposed** | | **separate** | | **proposed** | | **separate** | |
| **Case** | **Scen** | **MSE** | **Var** | **MSE** | **Var** | **MSE** | **Var** | **MSE** | **Var** | **MSE** | **Var** | **MSE** | **Var** | **MSE** | **Var** | **MSE** | **Var** |
| 1 | 1 | 3.937 | 2.747 | 9.530 | 9.043 | 3.861 | 2.497 | 4.014 | 3.825 | 1.323 | 1.119 | 1.430 | 1.348 | 0.628 | 0.597 | 0.727 | 0.710 |
|  | 2 | 4.389 | 3.231 | 9.215 | 8.558 | 3.061 | 1.911 | 4.055 | 3.731 | 1.470 | 1.237 | 1.689 | 1.654 | 0.723 | 0.709 | 0.783 | 0.768 |
|  | 3 | 3.858 | 2.699 | 8.038 | 7.760 | 3.371 | 2.046 | 3.896 | 3.348 | 1.173 | 1.033 | 1.417 | 1.406 | 0.635 | 0.591 | 0.727 | 0.720 |
|  | 4 | 3.688 | 2.807 | 8.438 | 7.923 | 3.329 | 2.926 | 4.022 | 3.691 | 1.059 | 0.875 | 1.278 | 1.173 | 0.613 | 0.606 | 0.768 | 0.759 |
|  | 5 | 4.392 | 3.712 | 9.477 | 8.694 | 2.586 | 1.722 | 3.506 | 3.286 | 1.409 | 1.386 | 1.611 | 1.596 | 0.700 | 0.682 | 0.742 | 0.742 |
|  | 6 | 4.696 | 3.569 | 8.015 | 7.235 | 2.265 | 1.944 | 4.352 | 4.020 | 1.279 | 1.227 | 1.435 | 1.388 | 0.513 | 0.486 | 0.734 | 0.730 |
| 2 | 1 | 8.575 | 7.273 | 340.539 | 338.211 | 7.531 | 6.458 | 130.831 | 130.110 | 4.175 | 3.866 | 45.474 | 45.084 | 2.586 | 2.298 | 17.385 | 17.329 |
|  | 2 | 9.706 | 7.193 | 256.072 | 253.787 | 9.840 | 7.046 | 103.762 | 103.161 | 4.088 | 3.816 | 57.162 | 57.016 | 2.258 | 2.067 | 19.669 | 19.645 |
|  | 3 | 7.673 | 5.293 | 305.764 | 304.095 | 7.367 | 6.105 | 118.197 | 117.973 | 3.537 | 2.522 | 48.329 | 48.018 | 2.183 | 1.940 | 21.026 | 21.011 |
|  | 4 | 10.470 | 7.225 | 327.050 | 326.643 | 8.419 | 7.720 | 126.134 | 123.695 | 4.067 | 3.567 | 58.373 | 58.248 | 2.295 | 2.181 | 20.685 | 20.604 |
|  | 5 | 9.206 | 5.968 | 268.423 | 267.928 | 10.239 | 9.835 | 117.410 | 116.225 | 3.930 | 3.104 | 53.435 | 53.164 | 2.406 | 2.302 | 14.178 | 14.136 |
|  | 6 | 9.928 | 6.510 | 299.031 | 298.472 | 8.636 | 7.052 | 109.010 | 108.276 | 4.098 | 3.486 | 64.976 | 64.298 | 2.069 | 1.957 | 19.213 | 19.191 |
| 3 | 1 | 5.136 | 2.957 | 210.082 | 209.259 | 4.516 | 3.805 | 102.254 | 101.663 | 2.247 | 2.082 | 41.970 | 41.908 | 1.373 | 1.296 | 19.450 | 19.435 |
|  | 2 | 6.642 | 4.783 | 249.051 | 248.347 | 3.869 | 2.785 | 92.952 | 92.355 | 1.901 | 1.675 | 37.963 | 37.485 | 1.043 | 0.976 | 16.288 | 16.275 |
|  | 3 | 6.770 | 4.590 | 189.134 | 188.787 | 3.484 | 2.750 | 93.470 | 92.812 | 2.437 | 2.055 | 43.060 | 43.013 | 1.381 | 1.298 | 18.979 | 18.957 |
|  | 4 | 5.358 | 3.089 | 155.287 | 154.631 | 4.549 | 3.471 | 109.289 | 108.794 | 1.908 | 1.403 | 30.327 | 30.122 | 1.513 | 1.381 | 16.485 | 16.470 |
|  | 5 | 5.430 | 3.918 | 238.925 | 237.280 | 3.541 | 2.760 | 109.406 | 108.872 | 1.986 | 1.769 | 53.426 | 53.406 | 1.266 | 1.166 | 17.709 | 17.691 |
|  | 6 | 5.961 | 3.233 | 227.782 | 226.285 | 4.961 | 3.308 | 101.947 | 100.905 | 2.416 | 2.159 | 37.098 | 38.822 | 1.195 | 1.096 | 17.210 | 17.197 |

Appendix II: Analysis using the R program

To facilitate utilization of the proposed method by other researchers, we have developed an R program and made it publicly available at [www.github.com/shuanggema](http://www.github.com/shuanggema). Here we provide more details on data analysis using this R program. Data analyzed here are downloaded from SEER <http://seer.cancer.gov/data>. In what follows, R codes are presented in *italic*.

**1. Load the brain tumor data**

*load("brain.RData")*

The dataset has the following structure.


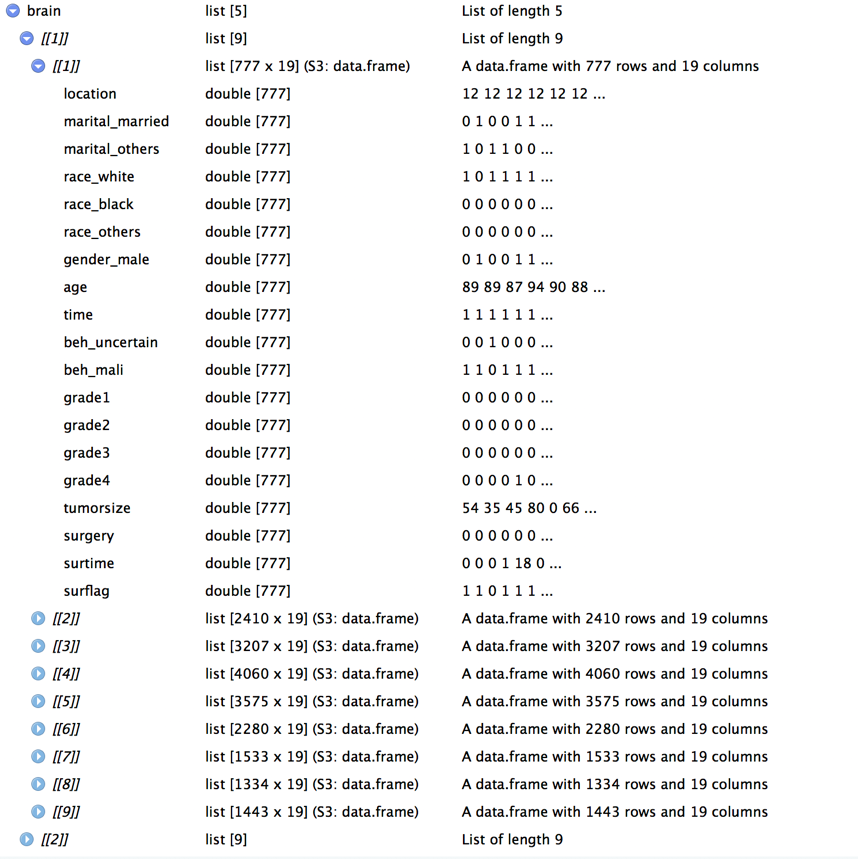


**Figure 4.** Structure of the brain tumor brain dataset in R.

2. Load R code for the proposed and separate estimations (and relevant functions)

*source("Functions.R")*

The following functions are available.


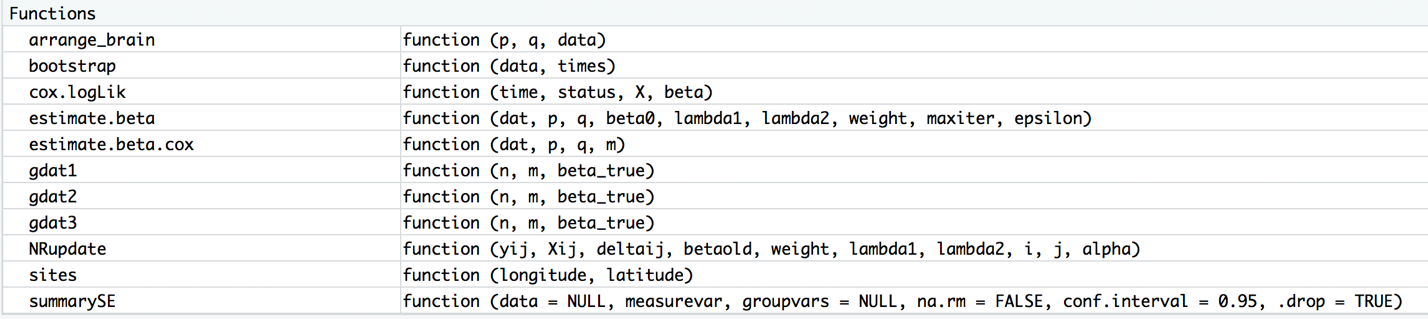


**Figure 5.** Functions loaded in R.

3. Set up parameters and weights

*m <- 15*

*p <- 16*

*q <- 9*

*maxiter <- 100*

*epsilon <- 0.001*

*longitude <- c(-122.41942,-72.661742,-83.0567,-157.796,-91.5299,-106.018066,-122.3301,-111.0937,-84.3880,-121.867905,-118.243683, -119.417931,-84.270020,-92.329102,-74.871826,-83.441162)*

*latitude <- c(37.77493,41.519813,42.3487,21.31139,41.6613,34.307,47.6038,39.3210,33.7490,37.279518,*

*34.052235,36.778259,37.839333,30.391830,39.833851,33.247875)*

*weight <- sites(longitude,latitude)*

*m* is the number of covariates, *p* is the number of locations, *q* is the number of time intervals, *maxiter* is the maximum number of iterations, and *epsilon* is the cutoff value to conclude convergence. *longitude* and *latitude* are the coordinates of each location. *weight* is the weights in penalization for pairs of locations.

**4. Survival analysis**

*realdat <- arrange_brain(p,q,data = brain)*

*beta_est <- estimate.beta(dat = realdat, p = p, q =q, beta0 = beta_cox, lambda1 = 40,*

*lambda2 = 1.2, weight = weight, maxiter = maxiter, epsilon = epsilon)*

*beta_cox <- estimate.beta.cox(dat = realdat, p = p, q = q, m = m)*

*beta_est* and *beta_cox* contain survival analysis results using the proposed and separate estimations, respectively.

**5. Bootstrap for inference**

*brain_unlist=brain[[1]][[1]]*

*for (i in 1:16) {*

*for (j in 1:9) {*

*b=brain[[i]][[j]]*

*brain_unlist=rbind(brain_unlist,b)*

*}*

*}*

*brain_unlist=brain_unlist[-c(1:195),]*

*bootresults <- bootstrap(data=brain_unlist, times = 50)*

*boot_cox <- bootresults [[1]]*

*boot_est <- bootresults [[2]]*

The above code conducts the nonparametric bootstrap for inference. *boot_cox* and *boot_est* contain results using the separate and proposed methods, respectively.

**6. Visualization of analysis results**

The following code demonstrates how to visualize the analysis results using marital status (married) as an example at each time interval and location=San Francisco-Oakland.

*location=1*

*cor=1*

*a<-rep(NA,30*q)*

*for (i in 1:30) {*

*a[(1+(i-1)*q):(q+(i-1)*q)]<-bootresult[[i]][location,,cor]*

*}*

*df <- data.frame(time=rep(1:q,30),Type=rep("Spatial− and temporal−smoothing",30*q),beta=a)*

*a<-rep(NA,30*q)*

*for (i in 1:30) {*

*a[(1+(i-1)*q):(q+(i-1)*q)]<-cox[[i]][location,,cor]*

*}*

*df1 <- data.frame(time=rep(1:q,30),Type=rep("Separate estimation",30*q),beta=a)*

*df2 <- rbind(df,df1)*

*df3 <- summarySE(df2, measurevar="beta", groupvars=c("Type","time"))*

*pp<- ggplot(df3, aes(x=time, y=beta, group=Type, color=Type)) +*

*geom_line() +*

*geom_point()+*

*geom_errorbar(aes(ymin=beta-ci, ymax=beta+ci), width=.2,*

*position=position_dodge(0.1)) +*

*labs(x = "Time",y="Beta")+*

*scale_x_discrete(limits=c("1","2","3","4","5","6","7","8","9"))*


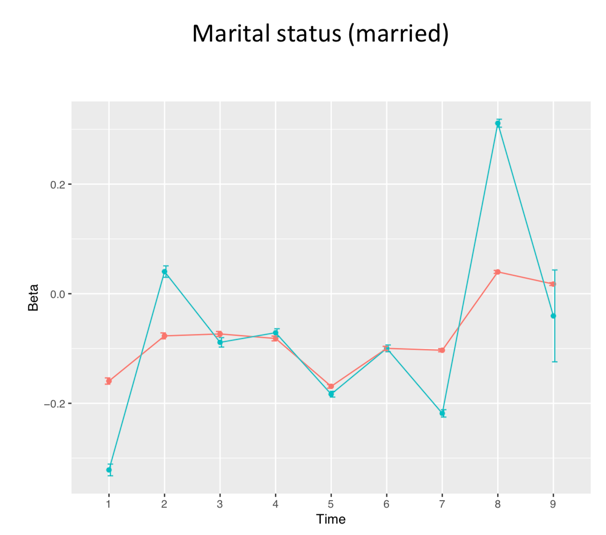


**Figure 6.** Visualization of an example of marital status (married) at each time interval and location=San Francisco-Oakland.

The following code graphically presents the analysis results for marital status (married) for the time interval 1911–1920 and each location.

*time=1*

*cor=1*

*a<-rep(NA,30*p)*

*for (i in 1:30) {*

*a[(1+(i-1)*p):(p+(i-1)*p)]<-bootresult[[i]][,location,cor]*

*}*

*df <- data.frame(location=rep(1:p,30),Type=rep("Spatial− and temporal−smoothing",30*p),Beta=a)*

*df1 <- summarySE(df, measurevar="Beta", groupvars=c("Type","location"))*

*a<-rep(NA,30*p)*

*for (i in 1:30) {*

*a[(1+(i-1)*p):(p+(i-1)*p)]<-cox[[i]][,location,cor]*

*}*

*df <- data.frame(location=rep(1:p,30),Type=rep("Separate estimation",30*p),Beta=a)*

*df2 <- summarySE(df, measurevar="Beta", groupvars=c("Type","location"))*

*df3<- rbind(df1,df2)*

*label<-c("San Francisco-Oakland","Connecticut","Metropolitan Detroit","Hawaii","Iowa","New Mexico","Seattle (Puget Sound)",*

*"Utah","Metropolitan Atlanta","San Jose-Monterey","Los Angeles","Greater California",*

*"Kentucky","Louisiana","New Jersey","Greater Georgia")*

*label<-data.frame(label,lon=locations[,1],lat=(locations[,2]))*

*text1<-data.frame(label=round(df1$Beta,3),lon=locations[,1],lat=(locations[,2]))*

*text2<-data.frame(label=round(df2$Beta,3),lon=locations[,1],lat=(locations[,2]))*

*us <- c(left = -162, bottom = 17, right = -67, top = 51)*

*usmap<-get_stamenmap(us, zoom = 5, maptype = "toner-lite") %>% ggmap()*

*pp<- usmap+*

*geom_point(aes(x=lon, y=lat, shape=Type,color=Beta),df3,size=2)+*

*geom_errorbar(aes(ymin=lat-ci, ymax=lat+ci,color=Beta), data=df3,width=.01,size=1.5)+*

*geom_text(aes(x=lon, y=lat, label = label),*

*data=label,*

*family = 'Times',*

*size = 3)+*

*geom_text(aes(x=lon, y=lat, label = label),*

*data=text1,color='red',*

*family = 'Times',*

*size = 3)+*

*geom_text(aes(x=lon, y=lat, label = label),*

*data=text2,color='blue',*

*family = 'Times',*

*size = 3)*


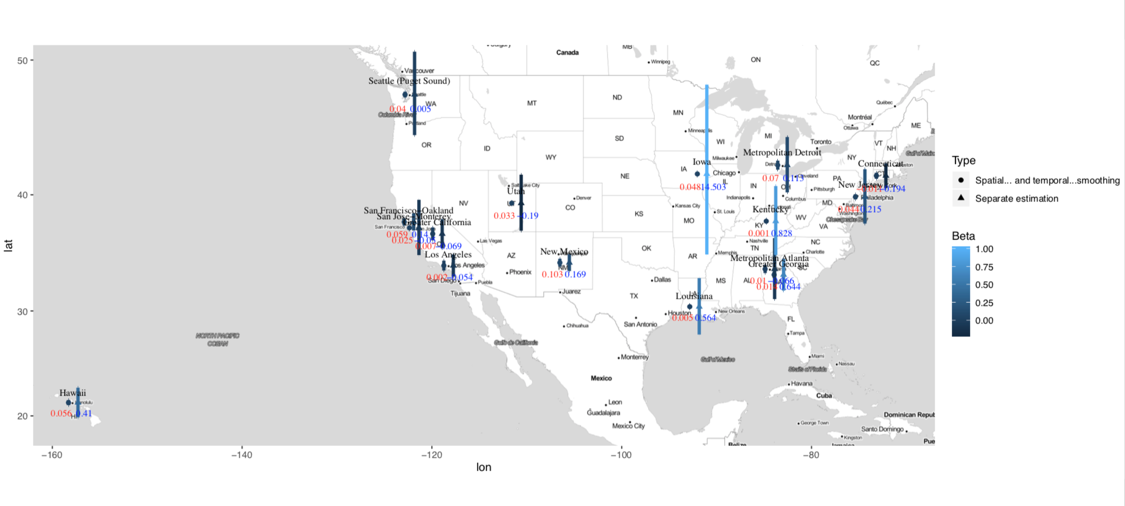


**Figure 7.** Visualization of an example of marital status (married) for the time interval 1911–1920 and each location.
